# Effect of Au thickness on the evolution of self-assembled Au droplets on GaAs (111)A and (100)

**DOI:** 10.1186/1556-276X-9-407

**Published:** 2014-08-20

**Authors:** Ming-Yu Li, Mao Sui, Eun-Soo Kim, Jihoon Lee

**Affiliations:** 1 College of Electronics and Information, Kwangwoon University, Nowon-gu, Seoul 139-701, South Korea; 2 Institute of Nanoscale Science and Engineering, University of Arkansas, Fayetteville, AR 72701, USA

**Keywords:** Self-assembled droplets, GaAs surfaces, Au thickness, Size and density evolution

## Abstract

In this paper, we report the effect of Au thickness on the self-assembled Au droplets on GaAs (111)A and (100). The evolution of Au droplets on GaAs (111)A and (100) with the increased Au thickness progress in the Volmer-Weber growth mode results in distinctive 3-D islands. Under an identical growth condition, depending on the thickness of Au deposition, the self-assembled Au droplets show different size and density distributions, while the average height is increased by approximately 420% and the diameter is increased by approximately 830%, indicating a preferential lateral expansion. Au droplets show an opposite evolution trend: the increased size along with the decreased density as a function of the Au thickness. Also, the density shifts on the orders of over two magnitude between 4.23 × 10^10^ and 1.16 × 10^8^ cm^−2^ over the thickness range tested. At relatively thinner thicknesses below 4 nm, the self-assembled Au droplets sensitively respond to the thickness variation, evidenced by the sharper slopes of dimensions and density plots. The results are systematically analyzed and discussed in terms of atomic force microscopy (AFM), scanning electron microscopy (SEM), energy-dispersive X-ray spectroscopy (EDS), cross-sectional surface line profiles, and Fourier filter transform (FFT) power spectra.

## Background

Self-assembled metallic droplets have been attracting considerable attention due to their outstanding physical and optoelectronic properties such as an improved optical absorption at their localized surface plasmon resonance (LSPR) frequency, the shift of wavelengths and the local heating, etc. through the interactions with quantum and nanostructures and thus have found various applications with diverse semiconductors. For example, self-assembled droplets can act as a nanoscale surface drilling medium for the fabrication of ‘nanoholes’ using the droplet etching technique [1-4]. Quantum dots have then been demonstrated around the nanoholes [5]. Also, metallic droplets have been successfully utilized in the fabrications of various quantum- and nanostructures such as quantum rings [6-9], quantum dots [10-12], and nanowires (NWs) [13] through ‘droplet epitaxy’ following the successful fabrication of homo-epitaxial GaAs nanocrystals on a GaAs substrate [14]. In addition, Au droplets have been adapted as catalysts for the fabrication of diverse NWs via various epitaxial approaches and have attracted extensive interest due to their unique properties such as surface plasmonic resonance, biosensing, quantum size effect, and biology [15-18]. Moreover, given the wide range of substrates and vapor phase materials utilized, Au droplets can be successfully utilized in the fabrication of various NWs and many elements utilized can diffuse into catalyst gold droplets based on the vapor-liquid-solid (VLS) mechanism during the fabrication of NWs [19-27]. For example, Si, Ge, GaN, GaAs, and InAs-InSb NWs have been successfully synthesized by molecular beam epitaxy, chemical beam epitaxy, pulsed laser deposition, and chemical vapor deposition [28-30]. In the VLS-based growth, from the supersaturated catalyst alloy droplets, the nucleation and growth of NWs can occur at the L-S interface due to a much higher sticking probability. Therefore, the design of NWs including diameter, length, configuration, and density is originally determined by that of the Au droplet catalysts. Consequently, the study of the behavior of Au droplets on various surfaces becomes an essential step to accomplish desired NW synthesis; however, to date, the systematic study of the control of Au droplets on GaAs is still deficient. Therefore, in this study, we investigate the effect of systematic thickness variation on self-assembled Au droplets on GaAs (111)A and (100).

## Methods

In this study, the fabrication of Au droplets was carried out on GaAs (111)A and semi-insulting (100) substrates in a pulsed laser deposition (PLD) system. The substrates used were epi-ready with an off-axis of ±0.1° from the American Xtal Technology (AXT, Inc., Fremont, CA, USA). Samples were initially indium bonded on an Inconel holder and degassed at 350°C for 30 min under 1 × 10^−4^ Torr in order to remove the contaminants. With the aim of investigating the effect of the Au thickness on the self-assembled Au droplets, various thicknesses of gold films were deposited at a growth rate of 0.5 Å/s with the ionization current of 3 mA as a function of time. The growth rate was calibrated by the XRD measurement. Gold films 2, 2.5, 3, 4, 6, 9, 12, and 20 nm thick were systematically deposited on GaAs (111)A and (100) at the same time in an ion-coater chamber under 1 × 10^−1^ Torr. Subsequently, substrate temperature (*T*_sub_) was ramped up to the target temperature of 550°C for an annealing process at a ramp rate of 1.83°C/s. The ramping was operated by a computer-controlled recipe in a PLD system, and the pressure was maintained below 1 × 10^−4^ Torr during the annealing process. To ensure the uniformity of Au droplets after annealing for 150 s, the *T*_sub_ was immediately quenched down to minimize the Ostwald ripening [30-32]. Subsequent to the fabrication of the self-assembled Au droplets, an atomic force microscope (AFM) was utilized for the characterization of surface morphology under the non-contact (tapping) mode with the AFM tips (NSC16/AIBS, μmasch). The Al-coated tips were between 20 and 25 μm in length with a radius of the curvature of less than 10 nm. The tip had a spring constant of approximately 40 N/m and a resonant frequency of approximately 170 kHz. The convolution of tips more sensitively affects the lateral measurement when measuring objects with high aspect ratios as well as high density in general. Thus, to minimize the tip effect and maintain consistency of the analysis, the same type of tips from a single batch were utilized for the characterization of Au droplets. The XEI software (Park Systems, Suwon, South Korea, and Santa Clara, CA, USA) was utilized for the analysis of the acquired data including AFM images, cross-sectional surface line profiles, and Fourier filter transform (FFT) power spectra. The acquired AFM images were processed by flattening along the *x* and *y* directions to improve the image quality. FFT power spectrum is generated by converting the height information from the spatial domain to the frequency domain using Fourier filter transform. Different colors represent different frequency intensities of height; thus, height distribution with directionality of nanostructures can be determined by the color distribution. For larger area surface characterization, a scanning electron microscope (SEM) under vacuum was utilized. The elemental analysis was performed using an energy-dispersive X-ray spectroscopy (EDS) system in vacuum with the spectral mode (Thermo Fisher Noran System 7, Pittsburgh, PA, USA).

## Results and discussion

**Figure 1:**
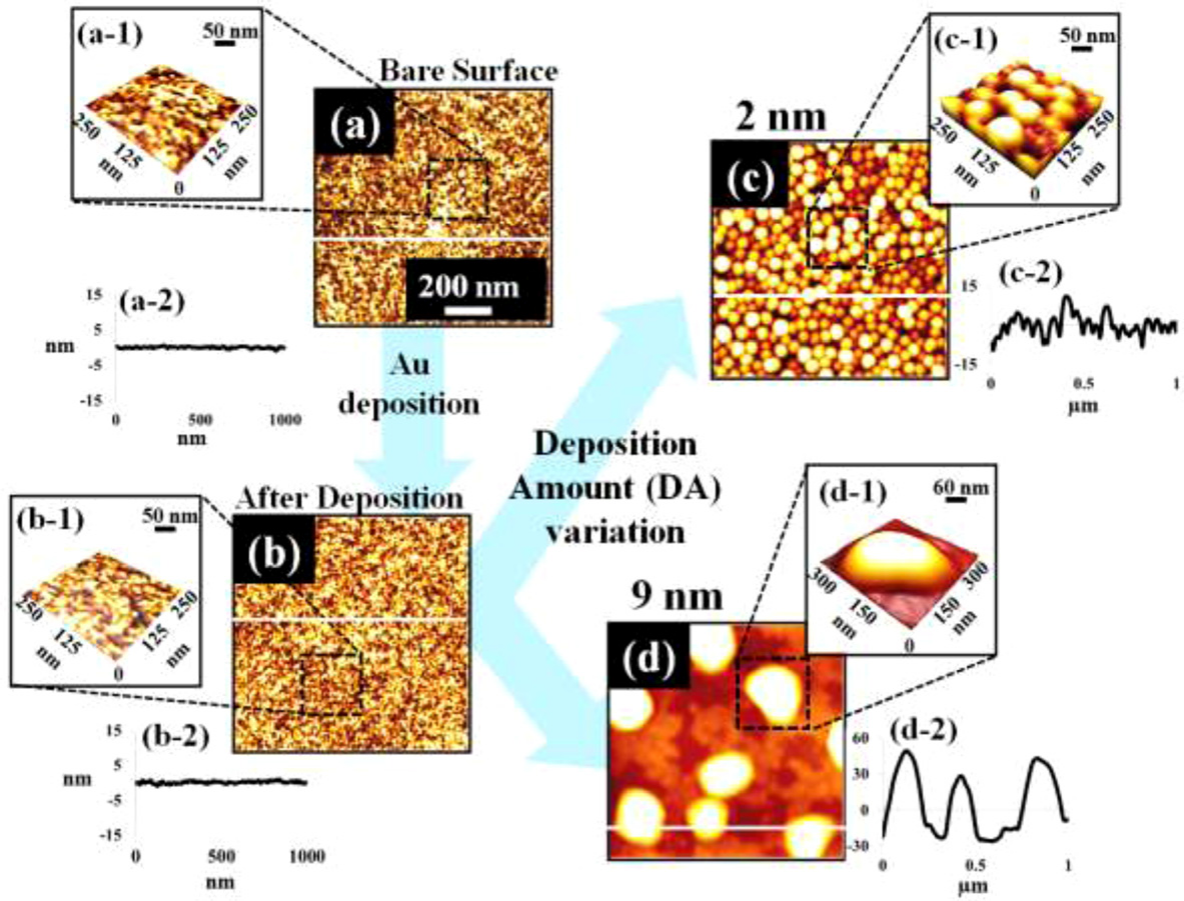
**Illustration of the fabrication process of self-assembled Au droplets according to the variation of Au thickness. (a)** Atomic force microscopy (AFM) image of bare GaAs (111)A. **(b)** After Au deposition. **(c)** Self-assembled Au droplets with 2 nm Au deposition annealed at 550°C. **(d)** Au droplets with 9 nm Au deposition. AFM images in **(a-d)** are 1 × 1 μm^2^. AFM side views of **(a-1)** to **(c-1)** are 250 × 250 nm^2^ and that of **(d-1)** is 300 × 300 nm^2^. **(a-2)** to **(d-2)** present cross-sectional surface line profiles indicated as white lines in **(a-d)**.

**Figure 2:**
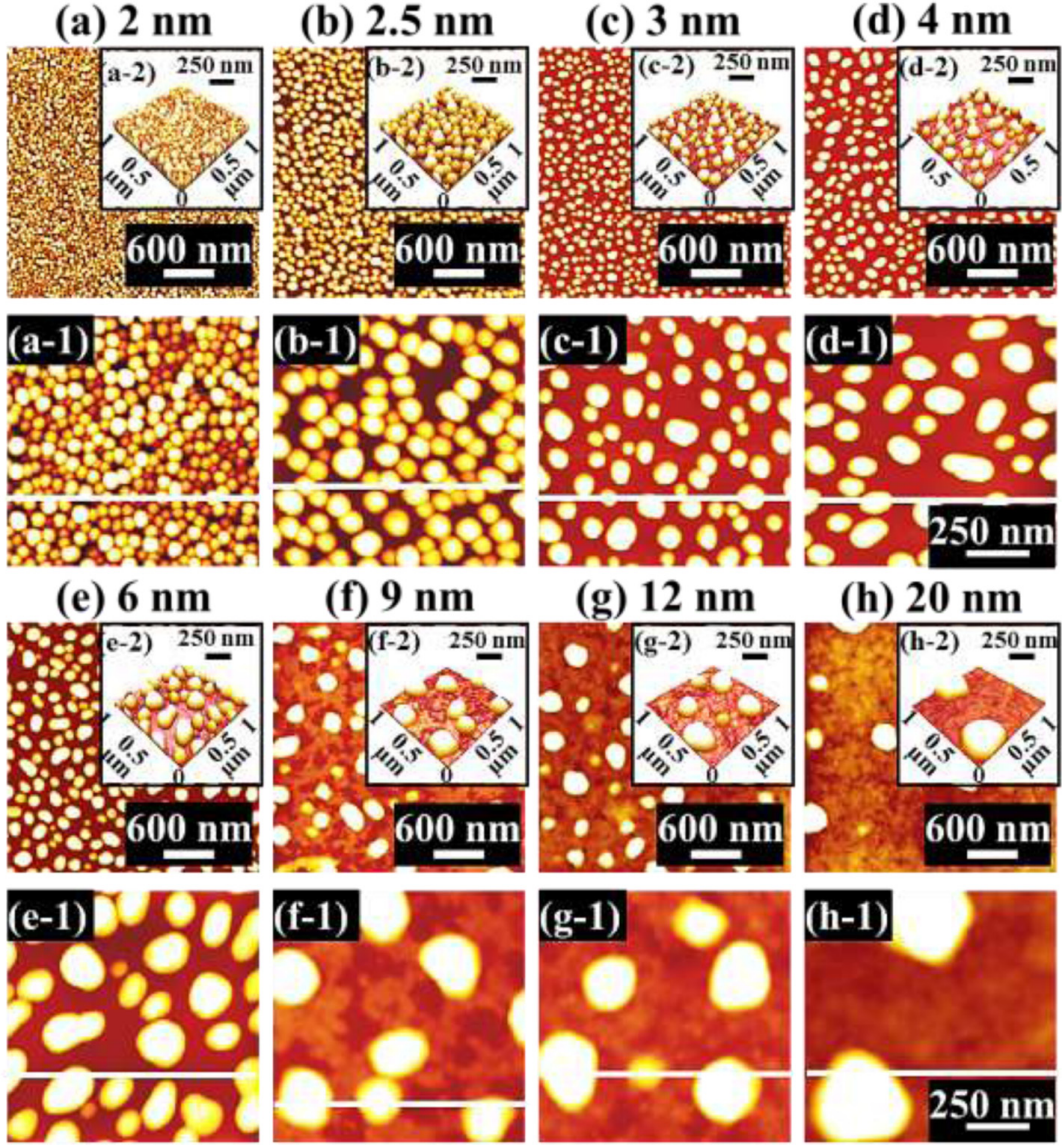
**Self-assembled Au droplets fabricated by the variation of the Au thicknesses between 2 and 20 nm on GaAs (111)A.** Au Droplets were fabricated by annealing at 550°C for 150 s. AFM top views of 3 × 3 μm^2^**(a**-**h)**. AFM top views of 1 × 1 μm^2^ [**(a-1)** to **(h-1)**]. AFM side views of 1 × 1 μm^2^ [**(a-2)** to **(h-2)**].

**Figure 3:**
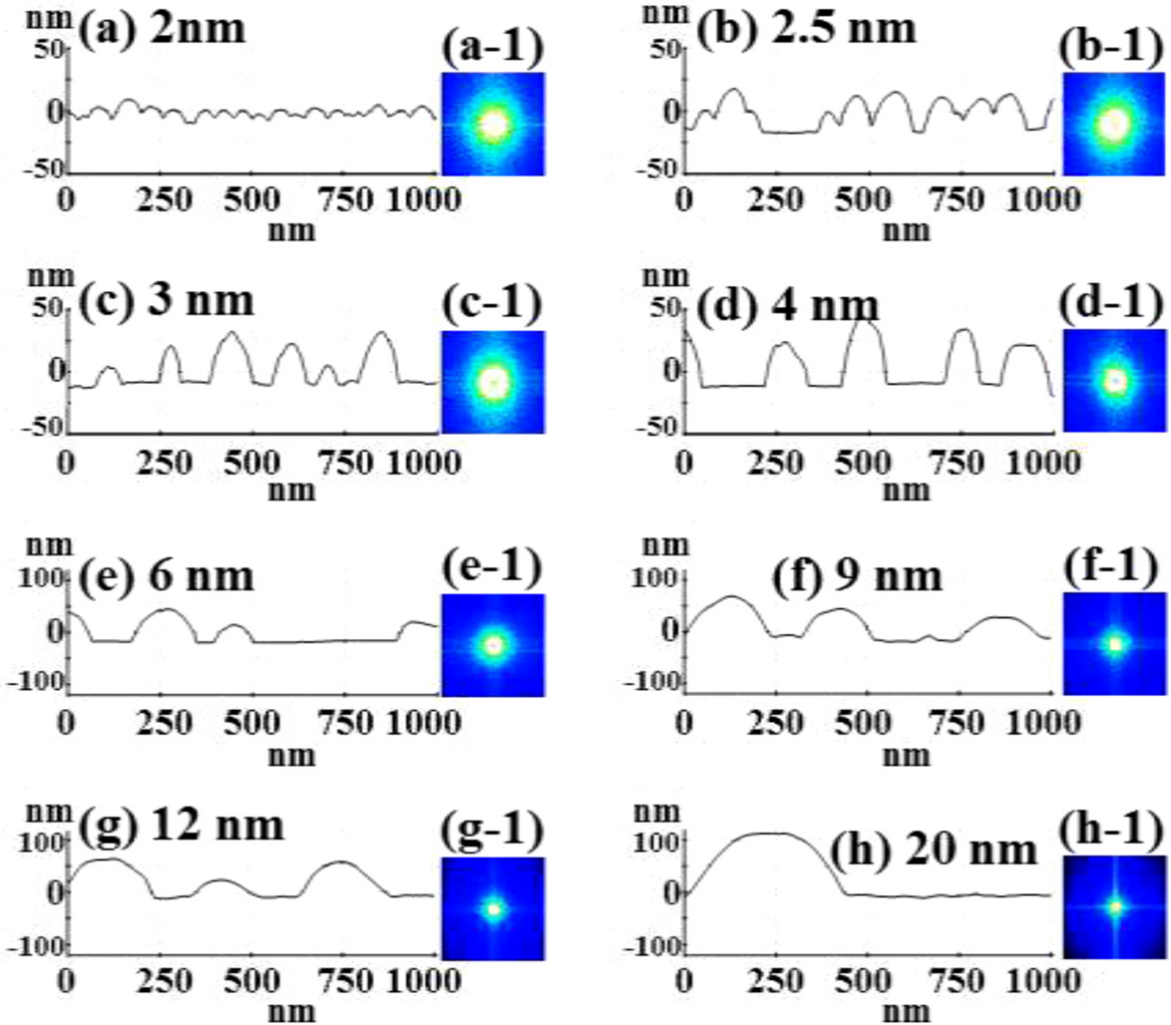
**Cross-sectional line profiles obtained from the white lines in Figure**2**(a-1) to (h-1) are shown in (a-h).** 2-D Fourier filter transform (FFT) power spectra of corresponding samples [**(a-1)** to **(h-1)**].

**Figure 4:**
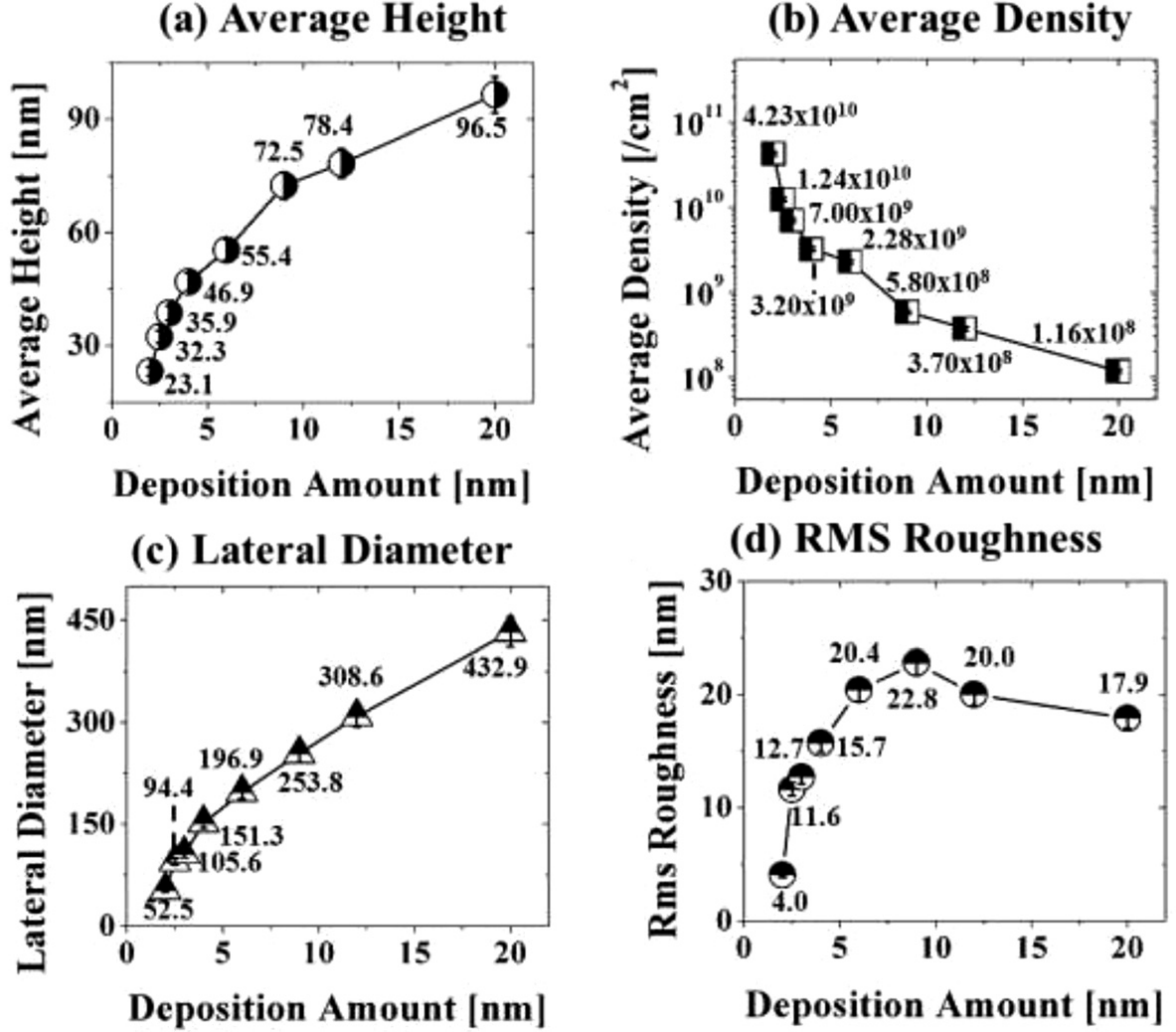
**Average height (AH), average density (AD), and lateral diameter (LD) of the self-assembled Au droplets.** AH **(a)**, AD **(b)**, and LD **(c)** of the self-assembled Au droplets fabricated on GaAs (111)A along with the Au thickness variation: 2–20 nm. **(d)** Root mean squared (RMS) surface roughness in nanometer of the corresponding samples. Error bars ±5% in all plots.

**Figure 5:**
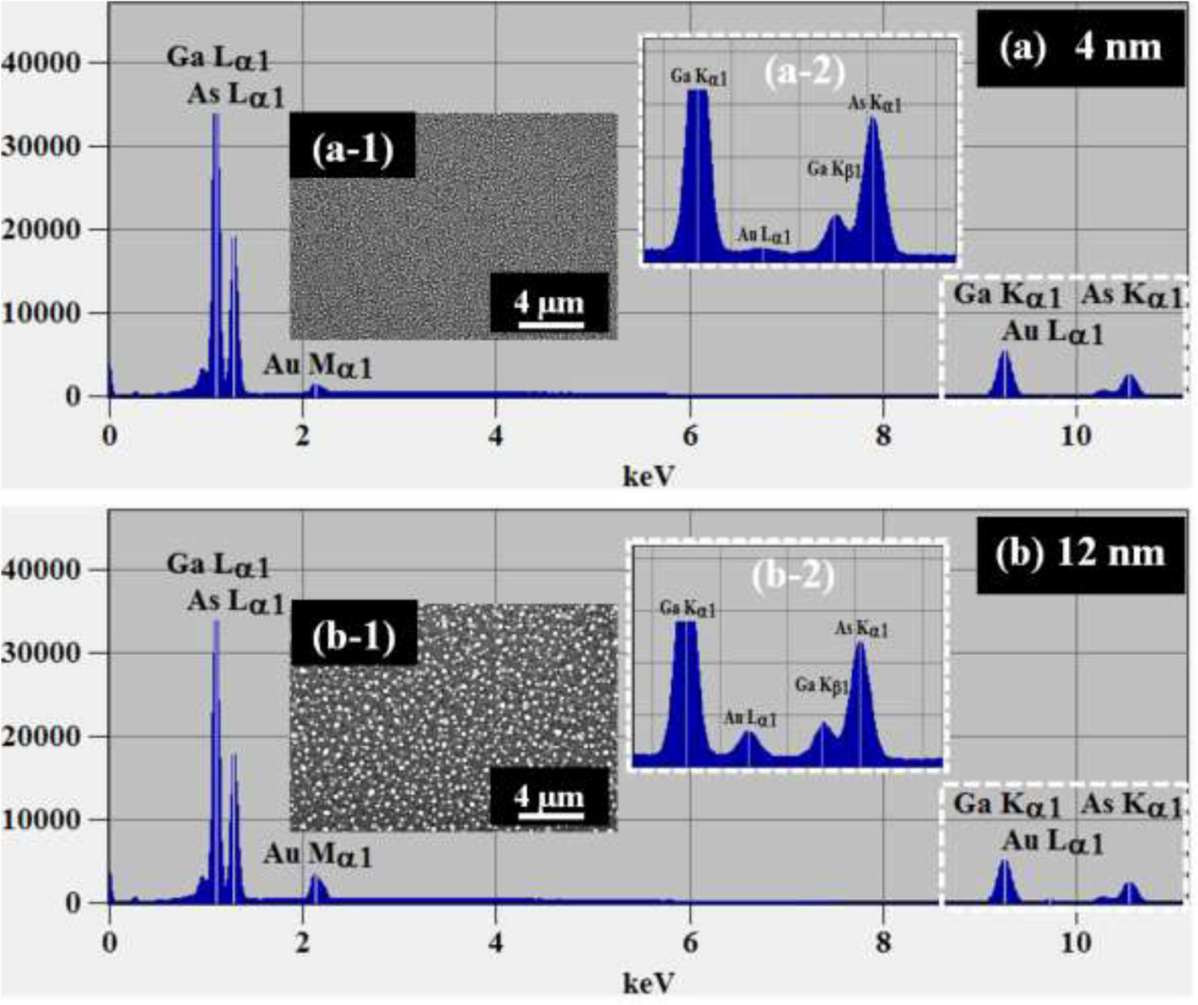
**Energy-dispersive X-ray spectroscopy (EDS) graphs.** EDS graphs showing the spectra of the samples with 4 nm **(a)** and 12 nm **(b)** Au thickness on GaAs (111)A. Insets in **(a-1)** and **(b-1)** show the corresponding scanning electron microscopy (SEM) images of a 20(*x*) × 13.88(*y*)-μm^2^ area. **(a-2)** and **(b-2)** show enlarged graphs between 9 and 11 KeV.

Figure 1 illustrates a simplified fabrication process of the self-assembled Au droplets on GaAs (111)A. For a systematic investigation, the Au film thickness (thickness) was carefully varied while fixing the other growth parameters. As clearly shown in the cross-sectional line profile in Figure 1(b-1), the surface was atomically smooth even after the Au deposition. The surface morphologies by a systematic annealing process are shown with 2 nm thickness in Figure 1c and 9 nm thicknesses in Figure 1d. Under an identical growth condition, the self-assembled Au droplets showed significant distinction in the size and density distribution depending on the thickness. Figure 2 shows the detailed evolution process of the self-assembled Au droplets on GaAs (111)A with the thickness variation between 2 and 20 nm. AFM top views of 3 × 3 μm^2^ are shown in Figure 2a,b,c,d,e,f,g,h, and those of 1 × 1 μm^2^ are shown in Figure 2(a-1) to (h-1). The insets in Figure 2(a-2) to (h-2) show the AFM side views of 1 × 1 μm^2^. Figure 3a,b,c,d,e,f,g,h shows the cross-sectional surface line profiles acquired from the 1 × 1-μm^2^ AFM images in Figure 2(a-1) to (h-1) indicated with white lines. FFT power spectra are shown in Figure 3(a-1) to (h-1). Figure 4 summarizes the average height (AH), average density (AD), and lateral diameter (LD) of the self-assembled Au droplets on GaAs (111)A compared to the various thicknesses. The root mean squared (RMS) roughness (*R*_q_) values of samples are summarized in Figure 4d. In general, the average size including height and diameter of the self-assembled Au droplets on GaAs (111)A was gradually increased with the increased thicknesses as clearly shown in the AFM images in Figure 2 and the surface line profiles in Figure 3 as well as the summary plots in Figure 4a,c. Meanwhile, the density of Au droplets was gradually decreased as clearly seen in Figures 2 and 4b. For example, with 2 nm Au deposition, the very densely packed dome-shaped Au droplets were formed on GaAs (111)A as presented in Figure 2a and (a-1) with the AD of 4.23 × 10^10^ cm^−2^. The corresponding AH was 23 nm and the LD was 52.5 nm as shown in Figure 4a,c. At 2.5 nm thickness, the size of droplets grew larger and the density was reduced as clearly shown in Figure 2b and (b-1): the AH was increased by × 1.4 to 32.3 nm and the LD increased by × 1.8 to 94.4 nm as shown in Figure 4a,c. On the other hand, as shown in Figure 4b, the AD decreased by × 3.41 to 1.24 × 10^10^ cm^−2^. With relatively lower coverage of 2 and 2.5 nm thicknesses, the Au droplets were quite round and uniformly distributed over the surface, as shown in the AFM images of Figure 2a,b. With 3 nm thickness, the Au droplets were also quite uniformly distributed over the surface and began to show a slight elongation as shown in the AFM images in Figure 2c. Similarly, with the further increase of thicknesses between 4 and 20 nm, the continuous decrease in density with the associated increase in size was clearly observed as shown in Figures 2,3,4. Overall, the size of Au droplets was increased by × 4.2 in AH and × 8.2 in LD between 2 and 20 nm Au deposition, and as a compensation, the AD was decreased by over two orders of magnitude as shown in Figure 4. Clearly, during the evolution of Au droplets, the lateral expansion was preferred and the size increase was compensated by the density decrease. The degree of increase in size and thus of the decrease in density was much pronounced at relatively thinner thickness such as below 6 nm as evidenced by the sharper slopes of the plots in Figure 4a,b,c. The expansion of droplet dimensions is also clearly observed in the RMS roughness (*R*_q_) plot in Figure 4d. With 2 nm thickness, the *R*_q_ was 4 nm and it was very sharply increased to 11.6 nm with only a slight increase of thickness to 2.5 nm. Then, the *R*_q_ was 12.7 nm with 3 nm thickness and 15.7 nm with 4 nm thickness. The *R*_q_ was then saturated at 9 nm with the maximum value of 22.8 and began to decrease, possibly due to the dominance of the density decrease. In terms of the shape of the Au droplets on GaAs (111)A, at relatively thinner thicknesses between 2 and 3 nm, the droplets showed a round geometry as clearly seen in Figure 2a,b,c, which were reflected in the FFT spectra in Figure 3(a-1) to (c-1) with the bright round patterns. Between 4 and 20 nm thicknesses, the Au droplets showed irregular shapes; however, the FFT spectra in Figure 3(d-1) to (h-1) remained round and symmetric as there was no specific directionality of elongation along any direction. The FFT spectra became dimmer due to the density reduction with the increased thicknesses. Figure 5 shows the EDS graphs with the thicknesses of 4 and 12 nm on GaAs (111)A. The insets of Figure 5(a-1) and (b-1) show the SEM images of the corresponding samples, and those of Figure 5(a-2) and (b-2) show the enlarged graphs between 9 and 11 KeV. In Figure 5a,b, identical Ga and As peaks are observed: the Lα1 peaks of Ga and As at 1.096 and 1.282 KeV and the Kα1 peaks of Ga and As at 9.243 and 10.532 KeV. Specifically, significantly pronounced Au peaks were observed with the 12-nm-thickness sample. For example, the Au Mα1 peak count at 2.123 KeV was nearly three times higher than that with the 4 nm thickness. Similarly the Au Lα1 peak at 9.711 KeV also showed nearly three times higher peak count as clearly seen in the insets of Figure 5(a-2) and (b-2), possibly due to the increased interaction volume of Au with the X-ray. Overall, with the increased thickness, the size of self-assembled Au droplets on GaAs (111)A continued to increase and the density continued to decrease, compensating the size expansion with the decreased density. Especially, at lower thicknesses (below 4 nm), the Au droplets were more sensitive to thickness, as revealed by the sharper slope shown in the plots in Figure 4.

In this experiment, with the increased thicknesses, the Au droplets persistently developed into 3-D islands with the dimensional increase including the height and diameter along with the decrease in density. This can be explained based on the Volmer-Weber mode [31]. After the nucleation, due to the weaker binding energy between surface and Au adatoms (*E*_I_) than the binding energy between Au adatoms (E_A_), Au atoms have a tendency to form 3-D islands rather than a layer (*E*_A_ > *E*_I_). The size expansion of Au droplets with increased thicknesses can also be seen with a variety of metal droplets on various surfaces [32-38]. As is well known, the diffusion length (*L*_D_) can be expressed as $L_{D}=\sqrt{D_{S}t}$, where *D*_S_ is the diffusion coefficient and *t* is the residence time of the atoms. The *D*_S_ is a direct function of the surface temperature. In this case, as the annealing temperature (*T*_A_) was fixed for all samples, an identical *L*_D_ can be expected. Meanwhile, in a thermodynamic system, a larger surface area is preferred with the nanostructures in order to reduce the surface energy. Thus, with the presence of additional Au atoms within the fixed *L*_D_, droplets tend to absorb near the Au adatoms to increase the surface area, until reaching equilibrium provided with the condition of *E*_A_ > *E*_I_. Therefore, with the increased thicknesses with a favorable diffusion, Au droplets can keep expanding in size with the accompanying decrease in density when thickness was increased. Au droplets on polystyrene, polymethyl methacrylate [39], Si [40], and TiO_2_[41] were reported to grow initially in the Volmer-Weber mode; however, Au droplets began to coalesce and even form a layer when the critical thickness was reached. The critical radius (<*R*_C_>) [41,42] can be expressed as $<R_{C}{>}^{4}\approx \frac{D_{S}\gamma \;\Omega^{4/3}}{\mathrm{KT}}D_{C}$, where *γ* is the surface free energy, *Ω* is the Au atomic volume, and *D*_C_ is the critical amount. As can be seen, the < *R*_C_ > is a direct function of *Ω* and *D*_C_, and thus, while other parameters are fixed, we can expect a direct increase of < *R*_C_ > with the thickness increase. For example, Au droplets on Si (111) [37] evolved based on the coalescence mode growth with the increased thickness and began to show an early stage of coalescence mode at a thickness as low as 5 nm and showed a significant coalescence at approximately 10 nm. With the thickness of 20 nm on Si (111), the Au droplets almost formed into a layer. However, perhaps due to the strong dominance of the Volmer-Weber mode in this experiment on GaAs (111)A, the coalescence mode did not occur and the self-assembled Au droplets persistently developed into 3-D islands with the increased thicknesses.

**Figure 6:**
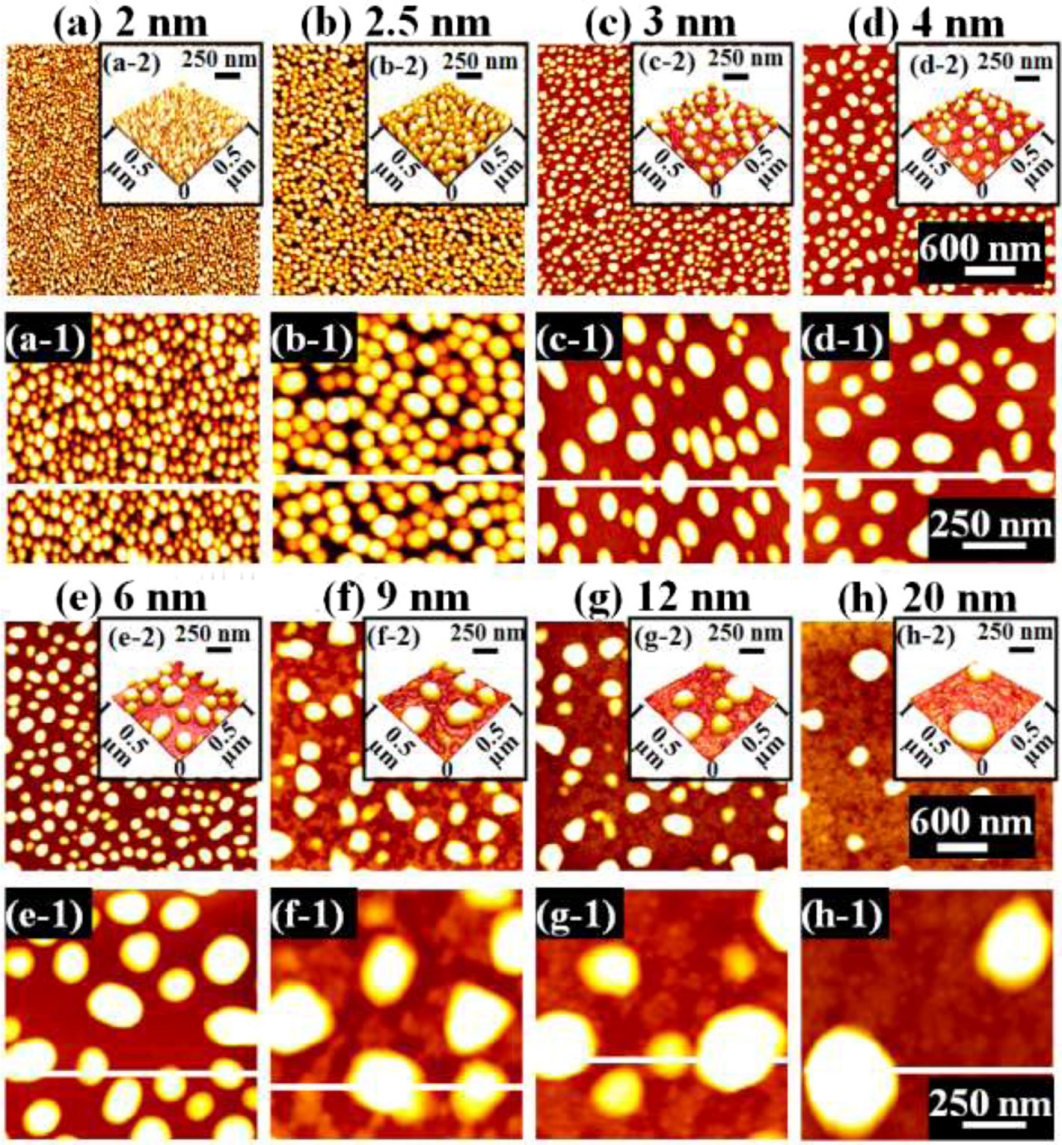
**Evolution of the self-assembled Au droplets.** Fabrication of Au droplets on GaAs (100) with the Au thickness, fabricated by annealing at 550°C for 150 s. The results are presented with AFM top views of 3 × 3 μm^2^ in **(a-h)** and of 1 × 1 μm^2^ in **(a-1)** to **(h-1)**. Insets in **(a-2)** to **(h-2)** are AFM side views of 1 × 1 μm^2^.

**Figure 7:**
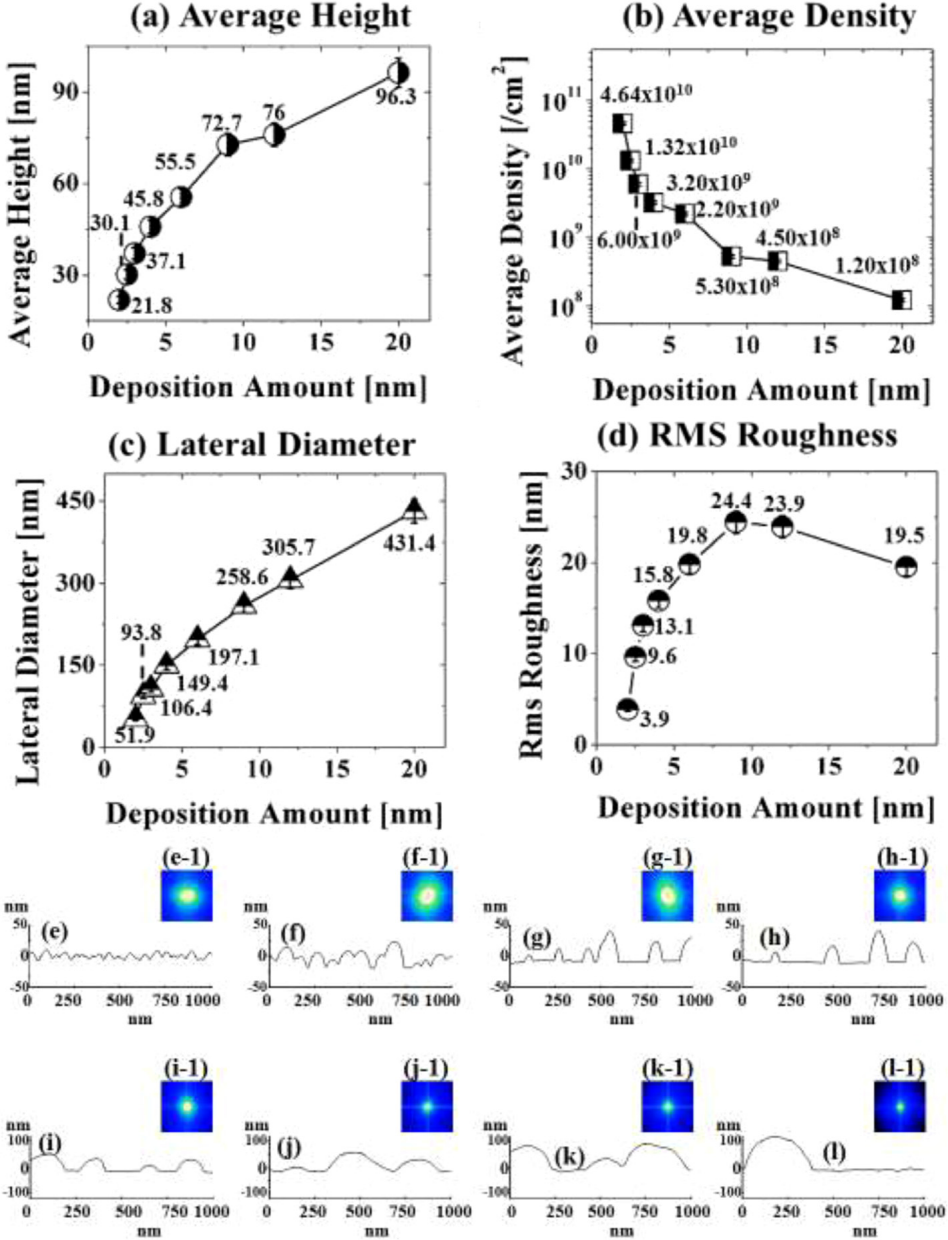
**Au droplet dimensions and RMS roughness.** Plots of Au droplet dimensions and RMS roughness on GaAs (100): AH **(a)**, AD **(b)**, LD **(c)**, and RMS roughness **(d)**. Self-assembled Au droplets were fabricated by annealing at 550°C for 150 s along with the variation of Au thicknesses (error bars ±5% in all plots.). Cross-sectional line profiles of Au droplets are shown in **(e-l)**, acquired from the white lines in Figure 6**(a-1)** to **(h-1)**. Corresponding 2-D FFT power spectra of each sample are shown in **(e-1)** to **(l-1)**.

**Figure 8:**
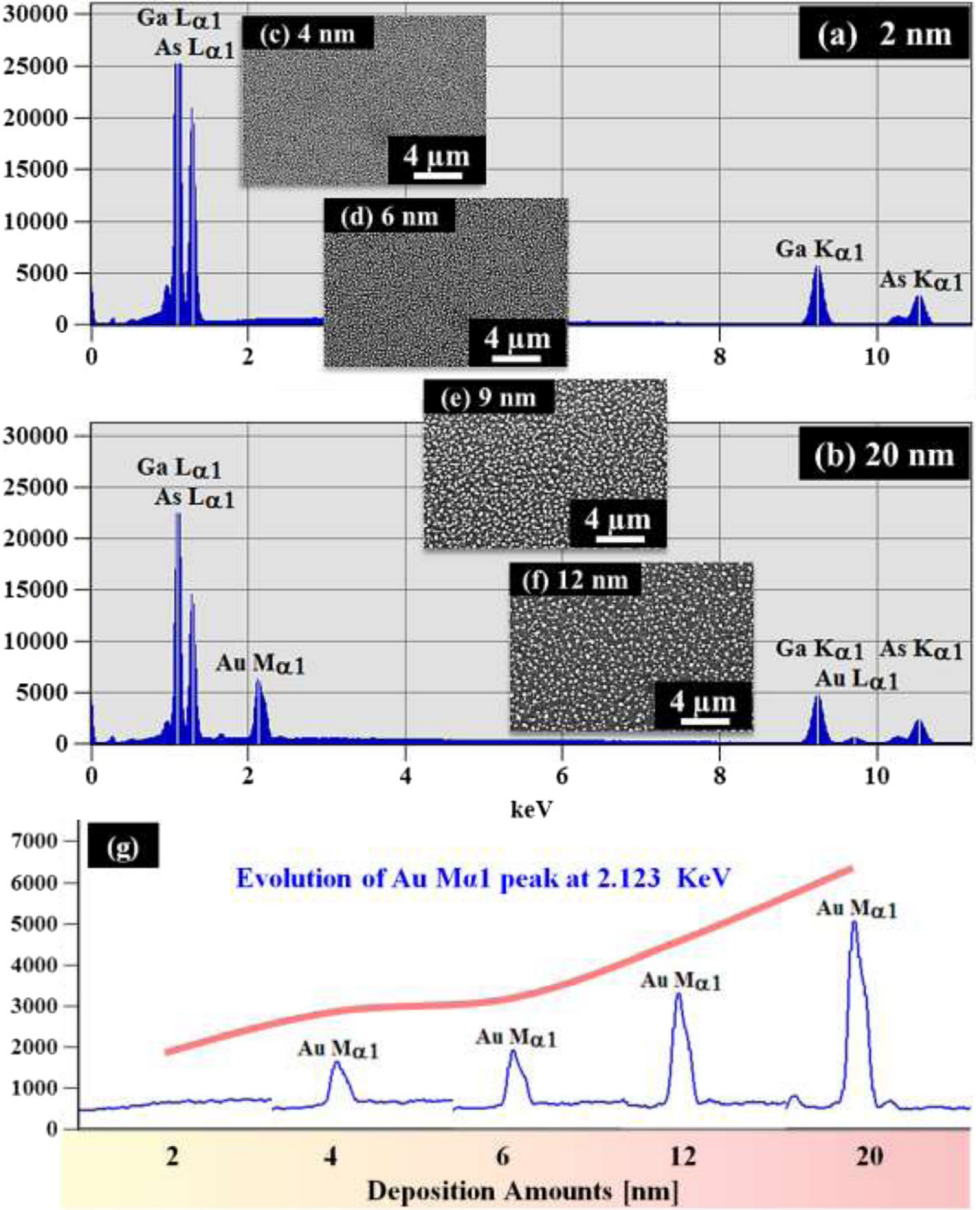
**EDS graphs of the samples with 2 nm (a) and 20 nm (b) thickness.** SEM images **(c-f)** reveal the size increase with decreased density of Au droplets at a larger scale. **(g)** Evolution of Au Mα1 peaks at 2.123 KeV along with the increased thickness between 2 and 20 nm.

Figure 6 shows the evolution of the self-assembled Au droplets on GaAs (100) along with the thickness variation between 2 and 20 nm, and Figure 7 summarizes the AH, AD, LD, and *R*_q_, as well as the corresponding surface line profiles and FFT power spectra, of the resulting Au droplets on GaAs (100). With 2 nm Au thickness, as shown in Figure 6a and (a-1), small dome-shaped Au droplets were formed with a packed high density. The corresponding AH and LD were 21.8 nm and 51.9 nm, respectively, as shown in Figure 7. The results were smaller droplets as compared to the droplets on GaAs (111)A by 5.63% in height and by 1.14% in diameter. Meanwhile, the AD was 4.64 × 10^10^ cm^−2^, 9.7% higher than those on GaAs (111)A. As the droplets were slightly smaller, the slightly higher AD can be accepted based on the diffusion and thermodynamics. The evolution of self-assembled Au droplets on GaAs (100) showed quite similar behaviors to that on GaAs (111)A in terms of the height, diameter, density, and *R*_q_ evolution as shown in Figure 7. That is, the size of the self-assembled Au droplets including the AH and LD gradually increased while the AD was progressively decreased when the thickness increased, as can be clearly seen in the AFM images shown in Figure 6 and the line profiles in Figure 7e,f,g,h,i,j,k,l. For example, at 2.5 nm thickness, the AH increased to 30.1 nm and gradually increased to 72.7 nm at 9 nm thickness, finally reaching 96.3 nm at 20 nm thickness as shown in Figure 7a. Similarly, the LD was increased to 93.8 nm at 2.5 nm thickness and finally reached 431.4 nm at 20 nm thickness. Meanwhile, the AD constantly decreased from 4.64 × 10^10^ cm^−2^ at the 2-nm thickness to 1.20 × 10^8^ cm^−2^ at the 20-nm thickness, as clearly seen in Figure 7b. The *R*_q_ was 3.9 nm with the 2-nm thickness and kept increasing up to 9 nm thicknesses with the value of 24.4 nm; it then began to decrease due to the dominance of density reduction in the evolution process. Overall, the size and density evolution of the self-assembled Au droplets showed a somewhat similar trend, and the size and density were also quite similar to those on GaAs (111)A. The FFT patterns shown in Figure 7(e-1) to (l-1) also show quite similar behaviors: round bright patterns with higher densities with thinner thicknesses, such as in Figure 7(e-1) to (h-1), and smaller patterns with reduced density with increased thicknesses, as shown in Figure 7(i-1) to (l-1). Figure 8 shows the EDS graphs with 2 and 20 nm thicknesses on GaAs (100), and the insets of Figure 8c,d,e,f show the SEM images of the samples with 4, 6, 9, and 12 nm thicknesses. Figure 8g summarizes the evolution of Au Mα1 peak at 2.123 KeV along with the increased thicknesses. The Au Mα1 peak at 2.123 KeV and Au Lα1 peak at 9.711 KeV were not observed in the large graph in Figure 8a, while the two Au peaks were clearly observed with the 20-nm thickness in Figure 8b. This could be due to the minimal interaction volume of the 2-nm-thickness sample. The SEM insets clearly show the size increase along with the decreased AD as a function of increased thickness, and Figure 8g clearly demonstrates the evolution of the Au Mα1 peak at 2.123 KeV as a function of increased thickness. In this work, the self-assembled Au droplets on GaAs (100) again showed quite similar evolution trends compared to those on GaAs (111)A. Based on the previous work [43], when the annealing temperature was varied between 250°C and 550°C on GaAs (100) and (111)A, respectively, the Au droplets showed a clear distinction in terms of their size and density. Indeed, at a lower temperature range between 250°C and 350°C, droplets began to nucleate and develop into wiggly Au nanostructures. Finally, between 400°C and 550°C, dome-shaped Au droplets were fabricated, and during the evolution, GaAs (111)A persistently showed larger-size Au droplets than GaAs (100). Meanwhile, GaAs (111)A constantly showed a lower density compared to the GaAs (100). Increased dimension of Au droplets was obvious with the increased annealing temperature based on the thermodynamics and diffusion perspective, as the *D*_S_ is a direct function of the surface temperature as previously discussed. With different surface indexes under an identical growth environment, the *L*_D_ can be affected by the root mean squared surface roughness (*R*_q_); this is caused by several factors such as the atomic step density, surface reconstruction, and dangling bond density [44-46]. The measured *R*_q_ values were 0.289 nm for GaAs (111)A and 0.322 nm for GaAs (100). Although GaAs (100) possesses a higher value of *R*_q_, the size and density between GaAs (111)A and (100) were quite similar within the error range. Perhaps, it could be because the ideal surface energy values of GaAs (100) and (111) are quite in a similar range: 65 meV/Å^2^ for GaAs (100) and 62 meV/Å^2^ for GaAs (111), respectively [47]. And also, it could be because the *L*_D_ of Au adatoms has a much more noticeable effect with the temperature variation based on the diffusion and the annealing temperature variation effect on various GaAs surfaces [43]. Namely, in this experiment, the size and density of Au droplets can be governed by thermal surface diffusion and the surface index can have a minor effect when the *L*_D_ was fixed with a fixed annealing temperature. Another possibility is that the difference is buried under the large degree of change in size and density induced by the thickness variation. For example, the AH of the Au droplets only varied by 23.4 to 32.4 nm when the annealing temperature was varied between 400°C and 550°C while the AH varied by 23.1 to 96.5 nm here when the thickness was varied between 2 and 20 nm.

## Conclusions

In conclusion, the evolution of self-assembled Au droplets on GaAs (111)A and (100) with a systematic variation of the Au thickness (thickness) between 2 and 20 nm has been investigated and the results were analyzed using AFM, surface line profiles, FFT spectra, SEM, and EDS data. The self-assembled Au droplets were fabricated based on the Volmer-Weber growth mode on GaAs (111)A and (100), resulting in distinctive 3-D islands, and the average dimension including height and diameter of the self-assembled Au droplets was gradually increased. While, the average density was progressively decreased along with the increased thicknesses on both GaAs (111)A and (100). The binding energy between the Au atoms is greater than that between the Au and surface atoms (*E*_A_ > *E*_I_); Therefore, the growth (even with the increased thickness) resulted in the formation of 3-D islands rather than a layer. At relatively lower thicknesses below 6 nm, Au droplets responded more sensitively in terms of the size and density evolution, shown by the sharper slopes of the size and density plots, which was also demonstrated by the sharply increased *R*_q_. The evolution of self-assembled Au droplets depending on the surface index showed quite similar behavior in terms of the size and density evolution. This can be due to the minor index effect when the diffusion length is fixed by the fixed annealing temperature; it could also be due to the excessive degree of change in the size and density of Au droplets. This result can be promising in various related nanostructure fabrications: quantum size effect, nanowires, biosensing, catalysis, study on the improvement of the localized surface plasmonic resonance, etc. on GaAs (111)A and (100) surfaces.

## Competing interests

The authors declare that they have no competing interests.

## Authors' contributions

M-YL, MS, and JL participated in the experiment design and carried out the experiments. M-YL, MS, E-SK, and JL participated in the analysis of data. M-YL, MS, and JL designed the experiments and testing methods. M-YL and JL carried out the writing. All authors helped in drafting and read and approved the final manuscript.
